# Molecular Dynamics Study of the Interaction of Carbon Nanotubes with Telomeric DNA Fragment Containing Noncanonical G-Quadruplex and i-Motif Forms

**DOI:** 10.3390/ijms21061925

**Published:** 2020-03-11

**Authors:** Tomasz Panczyk, Patrycja Wojton, Pawel Wolski

**Affiliations:** Institute of Catalysis and Surface Chemistry, Polish Academy of Sciences, ul. Niezapominajek 8, 30239 Cracow, Poland; wojtonpatrycja@wp.pl (P.W.); pawl.wolski@gmail.com (P.W.)

**Keywords:** i-motif, G-quadruplex, DNA, carbon nanotube, molecular dynamics, replica exchange

## Abstract

This work deals with molecular dynamics simulations of systems composed of telomeric dsDNA fragments, iG, and functionalized carbon nanotubes, fCNT. The iG contains 90 nucleotides in total and in its middle part the noncanonical i-motif and G-quadruplex are formed. Two chiralities of the fCNT were used, i.e., (10,0) and (20,0) and these nanotubes were either on-tip functionalized by guanine containing functional groups or left without functionalization. We proposed a dedicated computational procedure, based on the replica exchange concept, for finding a thermodynamically optimal conformation of iG and fCNT without destroying the very fragile noncanonical parts of the iG. We found that iG forms a V-shape spatial structure with the noncanonical fragments located at the edge and the remaining dsDNA strands forming the arms of V letter. The optimal configuration of iG in reference to fCNT strongly depends on the on-tip functionalization of the fCNT. The carbon nanotube without functionalization moves freely between the dsDNA arms, while the presence of guanine residues leads to immobilization of the fCNT and preferential location of the nanotube tip near the junction between the dsDNA duplex and i-motif and G-quadruplex. We also studied how the presence of fCNT affects the stability of the i-motif at the neutral pH when the cytosine pairs are nonprotonated. We concluded that carbon nanotubes do not improve the stability of the spatial structure of i-motif also when it is a part of a bigger structure like the iG. Such an effect was described in literature in reference to carboxylated nanotubes. Our current results suggest that the stabilization of i-motif is most probably related to easy formation of semiprotonated cytosine pairs at neutral pH due to interaction with carboxylated carbon nanotubes.

## 1. Introduction

The noncanonical DNA forms like i-motif [1,2,3], iM, or G-quadruplex, Gq [4,5,6], have attracted considerable attention mainly due to their switchable folding/unfolding property. That property depends on easily controllable external factors like pH or presence of some ions [7,8,9]. Combination of iM or Gq with carbon nanotubes, CNT, led to fabrication of novel functional nanomaterials or even platforms for digital information storage [10,11,12,13].

Interaction of carbon nanotubes with iM has also important biological implications. It was reported that carboxylated single walled carbon nanotubes can selectively induce human telomeric iM formation [14]. Moreover, it was reported that carboxylated CNT are able to inhibit telomerase activity both in vitro and in vivo [15]. That property was usually linked to the presence of G-quadruplexes in the telomeric region of DNA but not to the i-motif. Nonetheless, it was observed that selective stabilization of iM by carboxylated CNT led to telomere uncapping and removal of telomere binding proteins which finally caused DNA damage response [15]. However, the molecular mechanism of iM stabilization by the carboxylated CNT is still not fully understood. Moreover, the biological role of i-motif is far less recognized than the role of G-quadruplex, which is known as telomerase inhibitor [5,6]. It is believed that i-motif can exist only at reduced pH when the semi-protonated cytosine pairs CC+ can form [1,15,16]. Recent studies shed still more light into the biological role of iM. It was found that iM can also exist in regulatory regions of genome in living cells at physiological conditions [17].

Understanding the molecular mechanism of iM or generally telomeric part of the DNA interaction with CNT is important because the associated suppression of telomerase activity can be utilized in blocking of the infinite proliferative capacity of cancer cells. There are actually two possible ways for effective stabilization of iM by carbon nanotubes and they have already been outlined in literature [14]. The first way could be related to specific geometry of CNT and assumption that CNT can act as condensation nuclei to increase the likelihood of CC+ formation and indirectly leads to charge stabilization. The second way could be due to interaction between CC+ pairs and CNT, which lowers the pK_a_ of CC+ pairs protonation. Which of these two ways is more likely or dominant could be answered by application of dedicated theoretical studies, particularly molecular dynamics. However, until very recently there were no published results concerning the interaction of these noncanonical DNA forms with carbon nanotubes. First insights into a complex picture of an isolated iM interaction with functionalized carbon nanotubes, fCNT, were provided in our recent paper [18]. We found a few interesting features of iM adsorbed on the surface of fCNT but we did not observe selective stabilization of iM structure at neutral pH due to interaction with the fCNT. Conversely, we observed an enhanced destructive influence of a narrow (10,0) CNT on the iM spatial structure. Thus, we concluded that the stabilizing effect of carbon nanotubes is a more complex phenomenon, probably involving proton transfer reactions.

The aim of the current work is an analysis of a more biologically relevant case, that is, the case when the iM forms within the telomeric DNA duplex. In such a case, the complementary guanine-rich strand can fold into Gq structure because normally the necessary Na^+^ (or K^+^) ions are present in the solution. Thus, we subjected to careful theoretical analysis the telomeric DNA fragment consisting of 45 nucleotides in a single strand. In the middle of that dsDNA, we produced two noncanonical forms: iM and Gq and that complex structure, which we call iG, was subjected to interaction with two types of carbon nanotubes with chiralities (10,0) and (20,0). The nanotubes were on-tip functionalized by guanine residues or left without functionalization. The carboxyl functionalization was not applied because we first needed to understand how the presence of nanotube, without the possible proton transfer processes, interacts with the iG. The system turned out to be particularly difficult from a computational point of view. This is because bringing the overall system to thermodynamically optimal structure using a bias was accompanied by the destruction of the fragile iM and Gq fragments. Therefore, we had to elaborate a special procedure for that purpose and the detailed description of that procedure, which has a general applicability, is also an important part of this work.

## 2. Results and Discussion

### 2.1. Workflow in the Rigid Body Replica Exchange Simulations

The structures obtained from initial equilibration runs were subjected to additional treatment in order to find possibly most thermodynamically optimal configurations of fCNT and iG compounds. It is quite obvious that standard unbiased molecular dynamics runs lead to rather random configurations if the interactions within the system compounds are bigger than thermal energy. Thus, the configurations obtained from the initial equilibrations may be far from those thermodynamically optimal states. Therefore, we attempted several approaches in order to break local energy barriers and speed up the sampling of the whole configurational space. The first approach assumed the heating of the systems to high or even very high temperatures. However, it quickly turned out that fCNT and iG still reveal slow motion and application of high temperatures that mainly affect their internal degrees of freedom. It is not desirable, since the iG system and particularly its i-motif and G-quadruplex parts are quite fragile and heating them to temperatures significantly larger than the room temperature leads to their destruction. Obviously, we are interested in structures which can be reached by fCNT and iG at temperatures ca. 310 K. The enforced heating may lead to deteriorated and/or inaccessible configurations and this is not desired. Another approach applied to find an optimal thermodynamic configuration was the replica exchange method [19,20] (parallel tempering). Of course, that approach actually suffers from the same limitations as the direct heating since it involves a series of systems replicas running at higher and higher temperatures with the possibility of the temperature swaps between replicas. There was however another difficulty with the replica exchange approach. Namely, we found that temperature intervals between successive replicas must be quite small (ca. 3–4 K) because otherwise the temperature swaps did not occur due to very low probability generated by large potential energy differences. Thus, reaching temperatures which could lead to faster moves would require a lot of replicas and, in fact, enormous computational resources.

It was thus necessary to design a dedicated procedure which would be able to sample the configurational space effectively and with reasonable amount of computational resources involved. That procedure, which we call a rigid body replica exchange (rbREM), involves several steps which are presented here, using as a working example the system with (10,0) fCNT and the iG with protonated i-motif.

**Figure 1:**
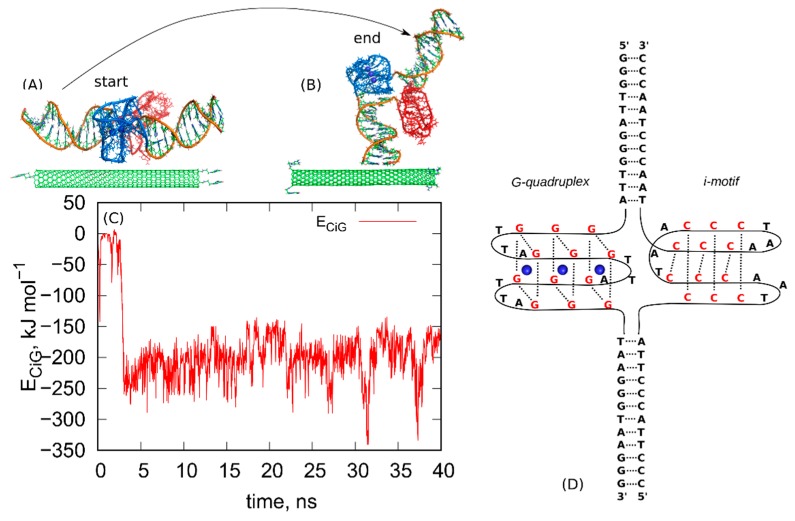
(**A**) The initial configuration of the system composed of (10,0) fCNT and protonated iG, (**B**) the final configuration obtained after 40 ns run in NPT ensemble. The blue parts are the G-quadruplex structures while the red ones are the i-motifs. The structures (**A**,**B**) are also provided as pdb files in the supplementary material: fig_1A.pdb and fig_1B.pdb. (**C**) The pair interaction energy between fCNT and iG as a function of time. (**D**) Schematic diagram showing the structure and nucleic acids sequence in the iG compound.

The first step in the rbREM is the equilibration run started from the initial configuration. That initial configuration is shown in Figure 1. As can be seen, we started from parallel alignment of fCNT and iG (Figure 1A) and some separation was applied between these two bodies in order to avoid overlapping. This a user-generated state and it quickly transformed to the state shown in Figure 1B. The mechanism of this transition is not important since it is probably driven by some initial strain in the system due to lack of full equilibration of the very initial atomic configuration. The calculations were continued for 40ns and we monitored the pair energy between fCNT and iG, *E_CiG_*. That energy is shown in Figure 1 as a function of time. It should be noted that the state in Figure 1B turned out to be highly stable, though probably far from thermodynamic equilibrium.

As can be seen, the *E_CiG_* quickly drops from the initial value ~0 to some new more negative value −250 kJ mol^−1^ and this corresponds to spontaneously reached closer contact between fCNT and iG. The *E_CiG_* does not change much until the end of the run and it only fluctuates around the mean value. The spatial configuration of the fCNT and iG also does not change much and is similar to that one shown in Figure 1B. This means that the system has been quickly trapped into a glassy state and further continuation of the unbiased calculations would not lead to a new state. The conclusion is that we have not got thermodynamically optimal state and there is no chance to escape from that state to another one using standard unbiased dynamics. To overcome that problem, we applied the following procedure. We took the last frame from the all-atom unbiased simulation run (Figure 1B) and used it as the initial configuration in the following steps.

(i) First, we transfered the system into a simple implicit solvent model. To that purpose we removed all water molecules from the system but left the saline ions. We replaced summation of the electrostatic interactions in the reciprocal space by a simple exponential decay of energy coming from point charges using Debye screening length and dielectric constant of water. Thus, the electrostatic part of the force field was computed using:

(1) $$E_{el}=\frac{q_{i}q_{j}}{\varepsilon r}\exp\left(-\kappa r\right)$$

where *E_el_* is the electrostatic interaction energy between point charges, *q_i_* and *q_j_*, ε is dielectric constant of water, is the Debye screening length in the electrolyte with the 0.145 mol L^−1^ ionic strength, and *r* is the distance between the point charges.

**Figure 2:**
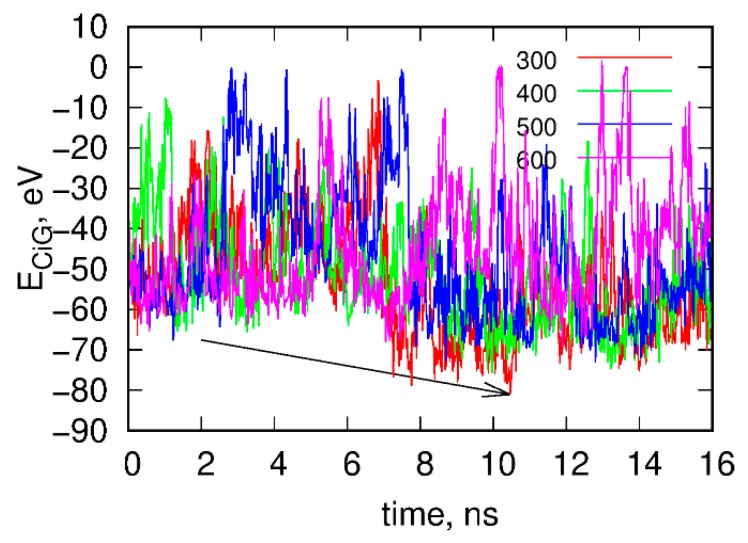
Time dependence of the interaction energy between fCNT and iG, *E_CiG_* in the rigid body replica exchange simulations. Note that *E_CiG_* does not match the energies from Figure 1. This is due to rescaling of ε*_ij_* by a factor of 0.1 and also to the transfer to the implicit solvent model. Each curve shows the energy obtained in a given replica from 300 to 600. The arrow shows the point when the interaction between fCNT and iG is the strongest. The configuration corresponding to this time point is next used as a starting configuration in the explicit solvent of all atom calculations.

We additionally rescaled all Lennard-Jones energy parameters, usually denoted as ε*_ij_*, by the same factor 0.1–0.2 in order to lower the whole potential energy landscape. So, the applied temperature range 300–600 K is enough for removing the system from any local potential energy well and thus we were able to sample the whole configurational space using a simple thermal agitation.

(ii) We transfered the classical point masses dynamics into rigid body dynamics. The whole fCNT and iG are then treated as two separate, but interacting, rigid bodies. The saline ions are still subjected to point masses dynamics. During this stage of computations, the integration was carried out in the NVT ensemble.

(iii) We ran rigid body replica exchange simulations rbREM with the rigid body dynamics applied to fCNT and iG. The simulations were carried out with four replicas corresponding to temperatures 300, 400, 500, and 600 K and the scaling factor for ε*_ij_* was adjusted in such a way that temperature swaps in rbREM occurred with a reasonable frequency. Figure 2 shows typical results obtained from the rigid body rbREM simulations. Each curve labeled 300, 400, 500, or 600 shows the *E_CiG_* energy obtained in a given replica. They normally do not correspond to temperatures of the replicas since the temperatures migrate between replicas.

The point denoted by the arrow in Figure 2 corresponds to the strongest interaction energy between rigid fCNT and rigid iG observed in rbREM simulations and the spatial arrangement of fCNT and iG corresponding to this point is the lowest energy configuration. Because the configurational space in rbREM is sampled very efficiently we can assume that this point represents the deepest minimum in the system potential energy, which is actually governed by the *E_CiG_* energy.

(iv) The lowest energy configuration is next taken as the starting configuration in the explicit solvent calculation. We simply inserted suitable amount of water molecules to the fCNT-iG system, restored the summation of electrostatic interactions in the reciprocal space and airebo force field for the internal degrees of freedom of the CNT, and finally restored the original values of ε*_ij_*. We next ran standard NPT integration for 4 ns which leads to even more energetically favorable configuration due to restored flexibility of fCNT and iG, and in that way we deepened the local potential energy well of the system.

**Figure 3:**
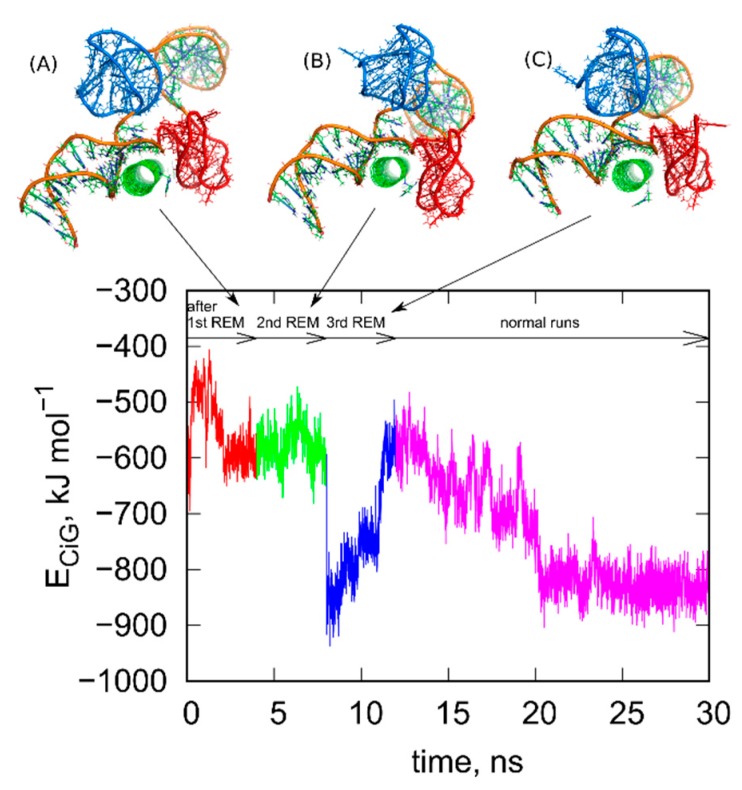
Changes of the *E_CiG_* energy after successive rigid body replica exchange (rbREM) calculations. The curve is divided into several sections corresponding to normal unbiased all-atom runs but interrupted by the rbREM periods. Each rbREM stage was done between time sections denoted by the short arrows. The snapshots (**A**–**C**) show system configurations found at the time points corresponding to the end of each short arrow. These configurations are available in the supplementary material as pdb files, files fig_3A-C.pdb.

Next, we repeated the steps (i)–(iv) several times until the configurations found in successive rbREM runs were the same or very similar. In the case of the studied system 3, successive repeats of the rbREM were enough. Figure 3 shows how the *E_CiG_* energy changes during each run following the rbREM stage and the configurations found at the end of each run. It should also be noted that the timescale in Figure 3 is not continuous. Simply prior to each section denoted by the short arrows in Figure 3, the rbREM runs from Figure 2 were performed (taking 16 ns per each replica) from which distinct fCNT-iG configurations are chosen. Continuous trajectory is produced only in the stage denoted by the long arrow (normal runs).

As seen in Figure 3, the *E_CiG_* energy changes in quite non monotonic way with however a clear tendency of getting lower and lower values that is stronger and stronger attraction between fCNT and iG. The energies in Figure 3 should be compared to the value from Figure 1. It is clear that after the first rbREM period, the *E_CiG_* energy rapidly dropped from ca. −200 kJ mol^−1^ to ca. −500 kJ mol^−1^ and the spatial alignment of fCNT and iG changed totally. The configuration presented in Figure 1, obtained from a quite long but unbiased run, is definitely a random one. The binding of iG by its duplex end to the sidewall of fCNT (Figure 1) is rather strange and we can expect that it is rather far from the thermodynamic equilibrium. Indeed, the application of rbREM quickly led to a more reasonable configuration (Figure 2) with the fCNT inserted between Gq and iM in the junction between duplex and the noncanonical structures. At the same time, the sodium ions entrapped in the Gq structures have been lost because during rbREM the temperatures reached 600 K. So, without making the duplex, Gq, and iM structures rigid they would not survive normal REM calculations.

As can be seen, the next two rbREM stages did not change the configuration obtained in the first rbREM substantially though the *E_CiG_* energy dropped to −900 kJ mol^−1^ after the last rbREM. It, however, increased to ca. −500 kJ mol^−1^ in an unbiased run but this effect is related to energy fluctuation. Further continuation of the unbiased simulations led finally to stabilization of either the *E_CiG_* energy or the mutual alignment of fCNT and iG. We can thus assume that the structures obtained after 3 stages of rbREM followed by ca. 10 ns unbiased simulations represent thermodynamically optimal configurations.

### 2.2. Analysis of Optimal Configurations of fCNT and iG Constructs

**Figure 4:**
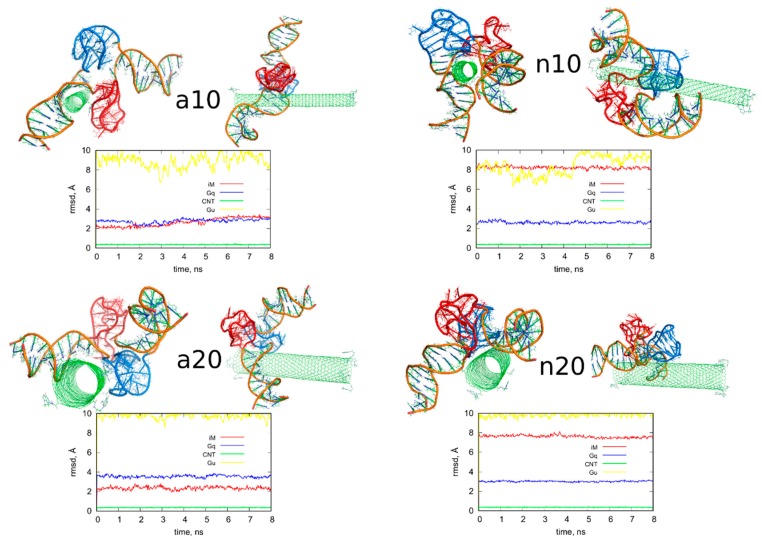
Simulation snapshots of the determined optimal configurations of fCNT and iG compounds and rmsd plots determined from the last 8 ns of simulations for iM, Gq, bare CNT and guanine functional groups (Gu). The G-quadruplexes are in blue color while i-motifs are in red color. Each of these configurations is available in the supplementary material as pdb files, files fig4_a10.pdb, fig4_a20.pdb, fig4_n10.pdb, fig4_n20.pdb.

**Figure 5:**
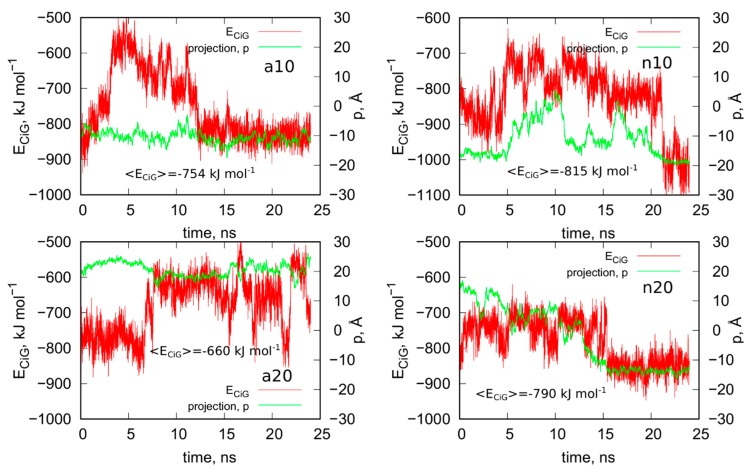
Interaction energy between iG and fCNT, *E_CiG_* as a function of time and projection of the center of mass of Gq and iM (taken together) into the CNT axis, *p*.

The above treatment was applied to the other three systems analyzed in this work. Figure 4 shows the obtained geometries after the rbREM runs, which are corresponding to the time point denoted by the end of the long arrow in Figure 3. However, in order to better understand the behavior of the iG structures interacting with fCNTs, we present in Figure 5 how the *E_CiG_* energies change in time. These energies are compared to the changes of the projection of the centers of mass of Gq and iM (taken together) into the nanotube axis. The meaning of the parameter *p* is simple; *p* = 0 means that the center of mass of Gq and iM is in the middle of the CNT and its change towards negative or positive values means that the iG moves to the left or to the right, respectively. Thus, analysis of *p* vs. *time* gives us a notion about the mobility of the iG in reference to the fCNT frame. Figure 5 also shows mean values of *E_CiG_* determined from the last 20 ns of the simulation.

We can now draw several general conclusions concerning the behavior of the iG and fCNT. Thus, we can notice that the iG always takes more or less resolved V-shape with the Gq and iM parts located at the edge of the V-shape. The ‘a’ systems reveal more open V-shape iG structures while the ‘n’ systems form more compact but at the same time more distorted V-shapes. Carbon nanotubes tend to fit between the arms of the V-shapes and it seems that iG structures prefer location close to CNT tips. We can also state that in ‘a’ systems, the iG compounds are rather static in reference to the nanotube frame (the projection p is almost invariant). In the ‘n’ systems, the iG is able to move on the nanotube surface though it still prefers to finally sit close to the nanotube tip. Another general observation is that the average interaction energy <*E_CiG_*> is bigger (its absolute value) in the ‘n’ systems. The structural changes of the systems are quantitatively illustrated in Figure 4 as the rmsd plots which were prepared using the last 8 ns of the simulations and the configurations from Figure 1A, as the reference states.

Let us analyze each system slightly closer. So, in the case of a10 system we can conclude that the iG structure is almost static and does not move on the CNT surface. This is because the projection of the center of mass of the Gq and iM is actually constant and these structures are quite far from the CNT tips, though one of the duplex parts of iG is at the fCNT tip. At the same time, we can see that *E_CiG_* energy changes substantially in time. This means that either the noncanonical parts of the iG do not contribute to that energy substantially or the Gq and iM are flanking without substantially changing their center of mass location, but with important changes related to the number of close contacts with the CNT surface. The second explanation is more likely, since in Figure 4 we can see that iM adheres to the CNT surface so its contribution to the *E_CiG_* energy cannot be negligible and Gq is indeed quite far away from the CNT. Table 1 confirms those observations and we can see that the most important contribution to the interaction energy comes from the interaction with the duplex part. It is clearly seen in Figure 4 (or in the provided pdb files) that fCNT binds to the major groove of the duplex and the iM and the duplex forms a circular tunnel within which the fCNT nicely locates. The immobilization of the iG is most probably due to interaction with the guanine functional group attached to the CNT tip. Closer analysis of this part of the system led to the conclusion that one of the guanines from the functional groups forms hydrogen bond with adenine (residue 51) from the duplex. That bond is not very strong since the distance between atoms forming hydrogen bond is 3.1 Å. However, it seems to be enough for immobilization of the iG since there are no other specific interactions (like stacking) with the CNT tip.

The second system with the protonated iM, that is a20, reveals similar property like a10, that is the iG is almost static on the CNT surface. However, it located on the opposite side of the CNT and the center of mass of Gq and iM is much closer to the CNT end than in the case of a10 system. The observed fluctuations of *E_CiG_* energy should also be assigned to flanking of Gq and iM because they do not move on the CNT surface. The interaction energy (Table 1) coming from Gq and iM is more than half of the total interaction energy with iG and the main component is in this case the interaction with Gq.

As seen, the wide (20,0) nanotube is located in the junction between the duplex and Gq and iM and tries to fit to the major groove of the duplex. However, in this case, due to the size of CNT, the area of contact of the nanotube and the iG is smaller than in the case of a10 system. A closer analysis of the distances between atoms belonging to the fCNT and iG leads to the conclusion that immobilization of the iG against shifts on the CNT surface comes from appearance of two hydrogen bonds between guanine functional group and iG. Precisely, these hydrogen bonds appear between nitrogen atoms from -NH and -NH_2_ in guanine and oxygen atoms belonging to phosphate backbone of adenine 69 residue. This residue belongs to iM part of iG and thus iM probably plays an important role in the immobilization of iG, though its contribution to the total *E_CiG_* energy is only about 1/4 of the overall *E_CiG_* energy.

The system with nonprotonated iM and (10,0) fCNT, i.e., n10, reveals qualitatively different behavior because in this case the center of mass of Gq and iM (projection, *p*) makes moves from the edge of the CNT towards its center and again approaches the fCNT tip. These moves are correlated with the changes of the *E_CiG_* energy, as seen in Figure 5. This energy is the highest (absolute value) among the studied systems and its large value is due to large number of close contacts of the fCNT with the iG. Especially contribution from iM is very large as seen in Table 1 but also interaction with the duplex plays an important role. Looking at the geometric configuration formed by fCNT and iG in Figure 4 (or analyzing pdb file), we can conclude that fCNT has been actually wrapped by the iG. We can also notice that one arm of the iG duplex sits on the fCNT surface and that the unfolded iM together with the major groove of the second arm of the iG duplex formed tubular tunnel where the fCNT can fit perfectly.

The final configuration shown in Figure 4 corresponds to the strongest interaction between iG and fCNT and we detected three distinct hydrogen bonds between iG and guanine functional group from fCNT. One of the hydrogen bonds is formed between guanine -NH and oxygen from the phosphate backbone, the other two hydrogen bonds form between nitrogen atom from adenine 62 residue and O and N atoms from guanine functional group. It seems that these hydrogen bonds are responsible for the *E_CiG_* energy drop at the end of calculations (between 21 and 24 ns) in Figure 5. Similar type and number of hydrogen bonds between iG and fCNT were also observed in the initial stage of calculations shown in Figure 5 (i.e., between 0 and 5 ns), but the structure of iM was less open than in the final stage. These hydrogen bonds of course disappeared when the center of mass of Gq and iM moved toward the middle part of the nanotube. Therefore, we can conclude that the appearance of those hydrogen bonds is not enough for making the iG immobilized at the fCNT tip. It, however, seemed to be enough in the case of systems with protonated iM, i.e., a10 and a20, so this problem needs further analysis.

The n20 system also reveals some correlation between position of the Gq and iM on the fCNT and the interaction energy, *E_CiG_*. As seen in Figure 5, the iG moved from one side of the nanotube to another during the first 15 ns and became immobilized. This is accompanied by the interaction energy drop but the binding is slightly weaker than in the case of the narrow nanotube, n10. In this case, the important part of the interaction comes from the Gq compound. Again, the guanine functional group from fCNT takes part in formation of hydrogen bonds with iG. We found two hydrogen bonds present in the final configuration of n20 system shown in Figure 4. One of them is formed between NH_2_ group from the guanine functional group and oxygen belonging to phosphate group linked to cytosine 58 residue. This residue belongs to iM part of the iG. The second hydrogen bond forms between oxygen atom from sugar group linked to adenine 57 residue.

The rmsd plots show how the internal structures of iM, Gq, CNT, or guanine functional groups change in time and in reference to their initial states. These plots are useful in illustration of the role of cytosines protonation and nanotube chirality in deterioration (or not) of DNA motifs. Thus, we can see that protonation of cytosines leads to stable i-motifs with rmsd not bigger than ca. 3 Å but deprotonation destabilizes these motifs as the rmsd grows up to ca. 8 Å. The effect is the same no matter of the nanotube chirality. G-quadruplexes are stable and are rather insensitive to protonation state of cytosines. However, we can notice some destabilizing effect of wide (20,0) nanotube on the structure of Gq. The rmsd of Gqs for (20,0) nanotubes are slightly higher than in the case of (10,0) nanotubes and grow above 3 Å. The nanotube cores are almost static and behave like rigid bodies since their rmsd is only slightly bigger than zero. On the other hand, the guanine functional groups changed their initial structures totally. They moved to the nanotubes sidewalls and this led to large rmsd values ca. 10 Å for these species.

**Figure 6:**
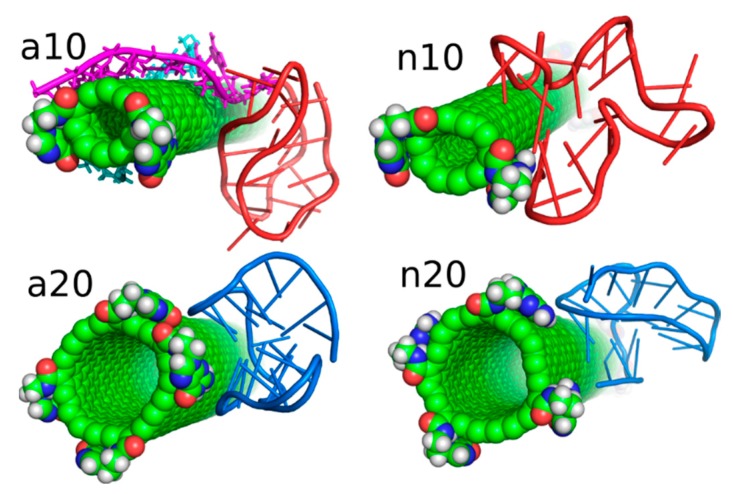
Architectural details of the essential interaction sites found in the considered systems.

As presented in Figure 5 and discussed in preceding sections, the iG adsorbs on the fCNT rather statically in all the studied cases. It means that in each case there exist some essential interaction areas or sites which contribute to the total interaction energy predominantly. These interaction sites can be detected looking at values shown in Table 1, i.e., combinations of pair interaction energies producing the highest energies (absolute values). Figure 6 provides graphical representations of these essential interaction sites. As seen in Table 1, the a10 system generates most of the interaction energy by the duplex part of iG with some small contribution coming from iM. Figure 6 shows this fragment of iG and fCNT just to expose architectural details of that configuration. In a20 system, the essential component of interaction is Gq part of iG. Gq essentially keeps it ‘basket’ shape but in the case of a20 system that shape is slightly deformed, as shown in rmsd plots in Figure 4. In n10 system, the strongest interaction comes from iM but at the same time the iM spatial shape is strongly deformed, as seen in Figure 6. Finally, in the n20 system, adsorption of Gq on the fCNT sidewall generates the strongest interaction and the ‘basket’ shape of Gq is well preserved upon adsorption.

The hydrogen bonds formed between guanine functional groups from fCNT and iG at places close to fCNT ends are not the typical Watson-Crick or Hoogsteen ones. They usually form between oxygen atoms from phospate backbones or sugars. Moreover, usually the angles between atoms are larger than the common value for hydrogen bond, i.e., 20deg. However, in every system the final and probably the most likely configuration is when the iG center (its Gq and iM area) approaches the fCNT end. It seems that the presence of guanine functional groups on the fCNT tips is the major reason of such configurations. Therefore, in the next part of the study, we re-analyzed the studied systems but with the guanine functional groups removed.

### 2.3. Analysis of Configurations of fCNT and iG Constructs after Removal of Guanine Functional Groups

**Figure 7:**
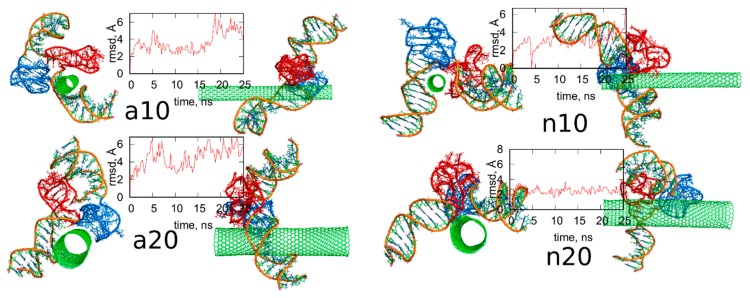
Simulation snapshots of the systems from Figure 4 after removal of guanine functional groups from fCNT and the rmsd plots of iG parts calculated for the next 25 ns of simulations. The reference configurations for calculation of rmsd were just the structures from Figure 4. The snapshots taken at the very last simulations timesteps. Each of these configurations is available in the supplementary material as pdb files, files fig7_a10.pdb, fig7_a20.pdb, fig7_n10.pdb, fig7_n20.pdb.

Figure 7 shows the simulation snapshots presented in the same way as in Figure 4. Visual comparison of the configurations shown in Figure 4 and Figure 7 leads to the conclusion that the lack of guanine functional groups has not changed significantly the structures of the iG adsorbed on the CNT surfaces. Additionally, the rmsd plots in Figure 7 for each case illustrates that the iG structures have not changed strongly during the whole 25 ns runs, with the reference structure being just the first frame. The rmsd were calculated for all atoms forming iG and their closer inspection, based on the analysis of the contributions from every residue to the total rmsd value, led to the conclusion that the prevailing components come from the displacements of terminal residues. In other words, the major component of the rmsd in each analyzed case come from flanking of duplex parts localized at the ends of iG. Interestingly, a slightly larger deformation of iG is observed in the cases of protonated iM, i.e., a10 and a20 systems where the rmsd oscillates around 5 Å. In these two cases, we also observed some deviations from the initial positions of the iM parts. Totally negligible changes in the iG structure after removal of guanine functional groups from fCNTs are observed for systems with nonprotonated iM, i.e., n10 and n20. The rmsd in these cases is ca. 3 Å, so these are not more than thermal fluctuations.

**Figure 8:**
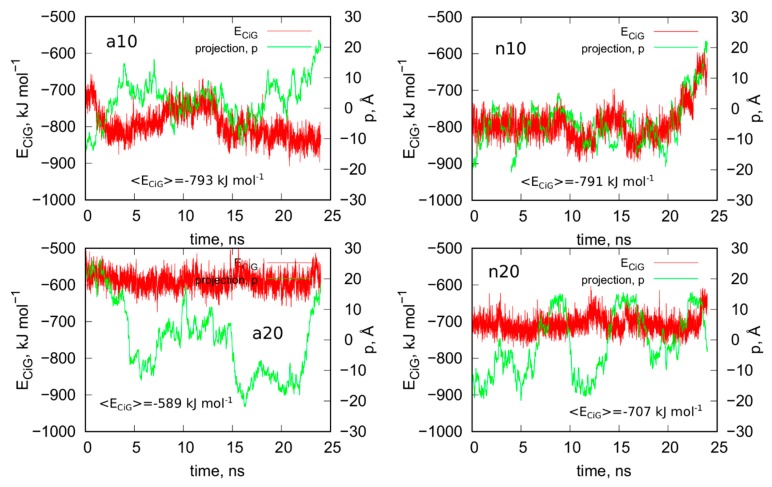
Interaction energy between iG and CNT for systems without guanine functional groups, *E_CiG_* as a function of time and projection of the center of mass of Gq and iM (taken together) into the CNT axis, *p*.

Figure 8 shows analogous results like in Figure 5 for systems without guanine functional groups. These are interaction energies *E_CiG_* and projections of Gq + iM centers of mass on the nanotube axis, *p*. The striking difference between Figure 5 and Figure 8 is that the projection *p* changes strongly during the run in the case of Figure 8. This means that iG moves freely on the CNT surface. The interaction energy however is almost constant, which means that it does not depend significantly on the position of iG in reference to CNT axis. There is one exception, i.e., n10 system, where the interaction energy weakens significantly when iG approaches CNT end. This however confirms the conclusion that the lack of guanine functional groups makes the iG highly mobile on the CNT surface. Of course, if iG would be connected to a larger DNA fragment then we could talk about CNT moving freely without any immobilization. This is a very important observation since we can use guanine functionalization in order to control how carbon nanotubes interact with iG containing DNA sequences. Incorporation of guanine residues to CNT tips leads to fCNT which can precisely attack Gq + iM part of DNA with its end (tip). The lack of guanine residues makes the CNT nonspecific and interacting with Gq + iM area by its sidewall. Thus, that observation can be useful in designing of smart delivery systems to these parts of the DNA.

The observed strong difference in behavior of fCNT and CNT without functionalized tips is quite unexpected since, as already discussed, the presence of guanine normally leads to the formation of only one or two hydrogen bonds with iG. Of course, there appear also extra Lennard-Jones interactions between more distanced atoms, but they normally are quite weak. Generally, the weakening of mean interaction energy <*E_CiG_*> after removal of guanine functional groups is between 3–11%, so these are rather small changes. We also did not observe any tangling of guanine residue and iG fragments, so this effect is also difficult to be explained by, generally speaking, entropy effects. Nonetheless the observation is very clear: guanine functional groups lead to attachment of fCNT tips to the junction between Gq and iM and DNA duplex.

### 2.4. Stability of i-Motif in iG Interacting with fCNT

A very important experimental observation related to the interaction of iM with fCNT is stabilization of iM structure at neutral pH by single walled carbon nanotubes [14,15]. This effect has also been addressed in our recent publication concerning the interaction of free iM fragment with fCNT [18]. However, the behavior of iM can be significantly different in the case of iG where iM is a part of a bigger structure. We therefore, applied biased simulations in order to measure works necessary to unfold the iM in controlled manner. The computational approach was quite similar to previously applied in the case of iM being a part of iG structure, but without the presence of fCNT [21]. Thus, we applied steered molecular dynamics, smd, and Jarzynski inequality [22] in order to estimate the free energy associated with the iM unfolding process.

A very important step in steered molecular dynamics is definition of a collective variable which properly describes the process under consideration. In this case, we took advantage of our previous experience with biased unfolding of noncanonical DNA fragments and defined the collective variable as the square root of mean squared displacement, rmsd, of atoms forming hydrogen bonds within the iM structure. These atoms can be easily identified looking at any graphical representation of iM, including the provided in the supplementary material pdb files. More illustrative schematic representation of these hydrogen bonds can be found in our recent publication [21] in Figure 1.

In smd, an external moving spring needs to be defined. This spring moves from the initial to the final value of the collective variable with a constant velocity and some value of the spring constant needs to be defined. In this case, we assumed that the spring moves from 0 to 25 Å rmsd with the velocity 1.25 Å ns^−1^ and the force constant was set to 5 eV Å^−2^. These parameters values were already verified as leading to good performance and accuracy of the smd calculations [21]. The reference states for calculation of rmsd were just the final states from unbiased calculations shown in Figure 4. However, they were taken directly only in the case of systems with protonated iM, i.e., a10 and a20. This is because the structures of iM in a10 and a20 systems were intact during the calculations. This was not the case in n10 and n20 systems, where iM were continuously deteriorating from the very beginning of the computations. The states in Figure 4 have passed about 100 ns in various stages of computations and thus the iMs in n10 and n20 system are already significantly deformed. Thus, calculation of works associated with the unfolding in biased simulations would lead to results which interpretation would be quite difficult. Instead we applied some tricks which should mimic the pH change from acidic to the neutral one, i.e., corresponds to situation when the stiff and highly ordered structure of iM formed at acidic pH is quickly transferred to neutral pH and quickly loosens protons attached to semiprotonated cytosines. This process corresponds technically to the replacement of the atomic structure of n10 and n20 systems by coordinates of atoms taken from the systems a10 and a20, respectively. All other parameters, including the force field topology, are of course preserved. So, in that way we can track the relevant factors, associated with the unprotonated iM, during the transition started from an ideal i-motif structure.

**Figure 9:**
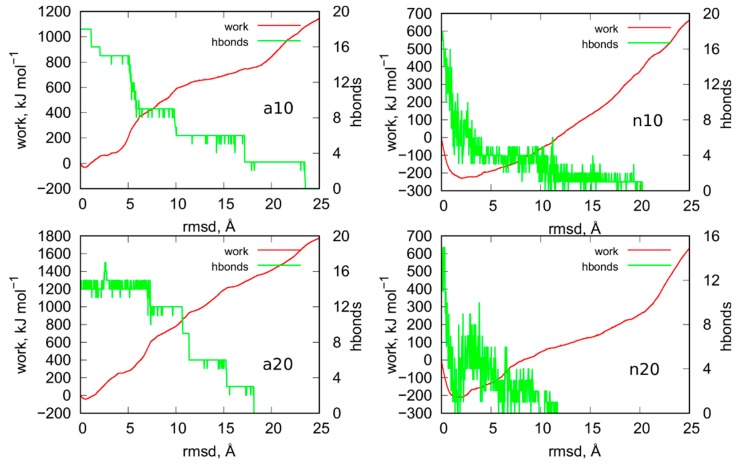
Works measured during the enforced unfoldings of iM parts of iG in biased steered molecular dynamics simulations. The biasing forces were imposed on atoms involved in formation of hydrogen bonds between C:C+ pairs and the moving restraints were imposed on the rmsd of given structures from the initial states. Green curves show how the number of hydrogen bond changes with the rmsd.

Figure 9 shows results from steered molecular dynamics, i.e., works done during the unfoldings of iM and also the number of hydrogen bonds between C:C+ pairs in iM at a given rmsd value. Looking at the curves *work* vs. *rmsd* for the systems a10 and a20 (protonated iM), it is difficult to state whether the size of the nanotube has really important meaning. Both curves have roughly the same shape and some differences between them are to be attributed to different initial configurations. It should be noted that in smd runs, the measured works versus collective variable differ also for very similar initial configurations. Therefore, in order to obtain more reproducible factors like potential of mean force (free energy), the exponential averaging of these curves is necessary [22]. However, in this case, similar to ref. [21], the averaging would be ineffective due to high computation costs of a single curve. Thus, we can safely conclude that the size of the nanotube has a rather minimal effect on the stability of the protonated iM. Moreover, the presence or not of a carbon nanotube has also a rather negligible effect on the stability of the protonated iM. This conclusion can be drawn by comparing the results in Figure 9 to the analogous results for iG system without the presence of nanotube. They have already been published in ref. [21] in Figure 6. As can be seen, the shapes of the curves are similar and also maximum values of works necessary to obtain 25 Å rmsd are in the range 1200–1800 kJ mol^−1^. Thus, in any of these cases, the protonated iM is highly stable.

The mechanism of unfolding of a10 and a20 system seems to be dependent on the nanotube size. We can notice that in a10 the initial number of hydrogen bonds is equal to the theoretical value, i.e., 18. It drops sequentially in groups of 3 (roughly speaking) because in C:C+ pairs we have always 3 distinct hydrogen bonds. In the case of narrow nanotube, a large value of rmsd ca. 24 Å is needed to break all hydrogen bonds. The a20 system reveals qualitatively different behavior because we can notice that at the beginning of the enforced unfolding 3 hydrogen bonds do not exist already. This effect must be attributed to the destructive influence of (20,0) nanotube on the structure of protonated iM which is surely very resistant. Visual inspection of this system leads to the observation that the (20,0) nanotube is able to destroy the bottom part of iM in the place where it connects with the canonical parts of the cytosine-rich strand. Finally, in a20 system we can observe that hydrogen bonds are breaking faster than in a10 case. However, the work necessary to break all of them is slightly bigger than in the case of narrow nanotube.

The behavior of n10 and n20 systems, i.e., the cases when the iG structure formed at acidic pH is transferred to the neutral pH, differs significantly from the cases a10 and a20. We can see that work done during the unfolding is negative until ca. 10 Å rmsd. It physically means that the process is spontaneous, and the moving restraint hinders the transition. Thus, the switch of pH from acidic to the neutral, which is associated with the change of the protonation state of cytosines, leads to very fast and spontaneous loss of iM symmetry. As seen in Figure 9, almost all hydrogen bonds quickly disappeared within 0–10 Å rmsd. It means that the Hoogsteen pairs C:C+ become unstable when one (of three) of the hydrogen bonds is removed. Then, the increase of the rmsd is associated with the change of C:C alignments from the very beginning. Contrary to the a10 and a20 systems where the C:C+ pairs were kept until the rmsd value 18–24 Å.

The effect of the nanotube diameter, i.e., the difference between n10 and n20 system, is rather small, like in the a10 and a20 cases. In both cases the work becomes positive for rmsd larger than ca. 10 Å which means that unfolding becomes slower than the speed of the moving restraint. However, the difference in stabilities of the systems with the protonated and unprotonated iM is clear. The protonated iM needs a lot of energy in order to displace atoms from the initial symmetry but the unprotonated one unfolds spontaneously. However, that spontaneous deterioration becomes at some moment slow or even stops and needs external energy supply to proceed further. A similar conclusion has been drawn in the case of iM being a part of the iG structure but without the presence of carbon nanotubes [23]. In those cases, the rmsd for the unprotonated iM slightly increased but after some time it reached a plateau. It physically meant that the initial structure of unprotonated iM became weakened but total deterioration of iM was stopped due to the presence of Gq or simply guanine-rich complementary strand. In turn, it is well known that free uprotonated iM unfolds spontaneously to a hairpin or random coil structures [7,24,25]. We have also already observed that the presence of carbon nanotubes actually speeds up iM deterioration [18]. The current results support the conclusion that unfolding of the unprotonated iM is hindered at some moment as the rmsd in Figure 7 of the whole iG structures is roughly constant in the considered periods of time. Thus, the unprotonated iM quickly loses hydrogen bonds between C:C pairs, reaches a more loose and open form, and stays in that form for a long time or even permanently. That static form of iM cannot, however, be called as regular i-motif since its symmetry is far from the perfect initial symmetry with 6 Hoogsteen C:C+ pairs. Nonetheless, due to presence of complementary guanine-rich strands, terminal fragments of cytosine-rich strands, and finally carbon nanotube, that form of iM becomes intact in time.

## 3. Methods

The applied methodology is based on the molecular dynamics simulations of model systems comprised of the functionalized carbon nanotubes fCNT and telomeric fragment of DNA, iG, in which the non-canonical Gq and iM are present. Both fCNT and iG have already been studied by us, but so far they have been analyzed separately. Thus, large parts of the methodology applied here, including generation of the simulation box topologies and force fields, are already available in the literature. This concerns the construction of the functionalized carbon nanotubes with all technical details, which is presented in ref. [18]. However, in this study we focused only on the guanine functionalized CNT with two chiralities (10,0) and (20,0).

The analyzed here telomeric fragment of DNA is the same as already described in refs. [21,23] iG model system. Shortly, this is the telomeric DNA fragment [5′-GGG(TTAGGG)_7_]: [3′-CCC(ATTCCC)_7_] where in the middle of the guanine-rich strand the G-quadruplex ‘basket’ topology is formed and in the middle of the cytosine-rich strand the i-motif is formed. Figure 1 shows a schematic diagram of the nucleic acids sequence within the studied iG compound. Two distinct version of iG were generated. The first one corresponding to neutral pH that is all cytosines were in their native forms. In the second version, half of the cytosines in iM were protonated, which formally corresponds to acidic pH. Technical details concerning the construction of iG with the discussion of the applied theoretical approaches is available in refs. [21,23].

The molecular topologies and force fields used for the iG and fCNT compounds are described thoroughly in our recent papers. Thus, details concerning the architecture of the DNA duplex, G-quadruplex, and i-motif are available in refs. [21,23]. The architecture of fCNT is, in turn, described in detail in ref. [18]. The new component of the force field concerns, however, the interaction of the fCNT with iG. Because the iG is described by the amber force field for nucleic acids (ff99bsc0+bsc1) [26,27], the obvious choice for mixed interaction between iG and fCNT was the generalized amber force field, gaff, designed for new drug molecules [28]. Thus, all carbon atoms within the nanotube are described as aromatic sp2 carbons and the guanine functional groups are described by the standard gaff force field. Internal degrees of freedom of carbon nanotubes were described by the airebo potential [29]. Therefore, the interaction between iG and fCNT is computed as pairwise additive sum of Lennard-Jones 12-6 contributions from all atom pairs between these two compounds. There is also a contribution from electrostatic interactions between point charges localized on iG atoms and also on fCNT atoms, though in the latter case most of carbon atoms forming CNT are charge neutral.

Taking into account that we consider two kinds of iG differing in protonation state of cytosines and two kinds of carbon nanotubes, differing in chirality, we have to deal with four different systems: a10, a20, n10, and n20. These are: protonated iM with (10,0) nanotube, protonated iM with (20,0) nanotube, unprotonated iM with (10,0) nanotube, and unprotonated iM with (20,0) nanotube, respectively.

The simulation boxes also contained adequate amounts of Na^+^ and Cl^−^ ions in order to compensate the charge of phosphate backbones and to mimic the ionic strength of solution 0.145 mol L^−1^. The number of water molecules was ca. 32,600 molecules, thus the simulation boxes contained about 102,000 atoms in total. The calculations were carried out using lammps code [30] in the NPT statistical ensemble with pressure and temperature controlled using the Nosé−Hoover barostat. A standard 12 Å cutoff distance for interatomic interactions was applied together with periodic boundary conditions. The electrostatic interactions were normally summed up by applying the particle–particle particle–mesh solver; however, during the rigid body replica exchange runs, the electrostatic interactions were calculated using Debye scheme as described in the next section. The total calculations times were divided into several stages; first stage took 40 ns of unbiased calculations started from initial configurations. Next, three repeats of 16 ns of biased rigid body replica exchange followed by 4 ns of relaxation were done, and finally 16 ns of production runs were done. Thus, the total calculation time for each system was 116 ns. These times were additionally completed by 20 ns of steered dynamics in order to measure the works necessary to unfold the i-motifs.

## 4. Summary and Conclusions

This work deals with two distinct but actually coupled important problems related to the interaction of carbon nanotubes with DNA fragments containing non-canonical DNA structures. The first problem is related to purely computational side and it was detected during attempts of determination of thermodynamically optimal configurations of fCNT and iG. It turned out that those optimal configurations are unreachable in standard unbiased calculations and application of various biasing methods, which should help to pass the energy barriers (infrequent events), destroys the fragile structures of the noncanonical iM and Gq forms.

Therefore, we proposed computational procedure which allows probing configurational space of two (or more) complex bodies efficiently and, at the same time, it keeps the fragile internal structures of those bodies unaffected by the biasing factors. This can be achieved by (i) transferring the essential components of the system to a simple implicit solvent model, (ii) rescaling the potential energy landscape so that the energy barriers can be overcome by the applied biasing factor, (iii) transferring the essential components of the system into rigid bodies and running computation according to the rigid body dynamics, (iv) applying biasing factors like increasing the simulation temperature and running the replica exchange simulations. This approach, as presented in Section 2.1, allows to quickly obtain configurations which are much closer to thermodynamically optimal ones than the configurations obtained from very long standard unbiased calculations.

By applying the above procedure, we were able to analyze and discuss the configurations of guanine functionalized fCNT and iG. We found that preferred alignments of these compounds is when fCNT locates in the junction of Gq + iM and the duplex and with the iG immobilized on the nanotube surface. The lack of guanine functional groups on the nanotube tips leads, however, to a free motion of iG on the nanotube surface. The general and important conclusion is that the presence of guanine functional groups favors configurations with the nanotube tips close to the Gq + iM section. In other words, such type of CNT functionalization makes the nanotubes able to selectively attack the Gq + Im parts of DNA with its tip. The lack of guanine functionalization leads to nonspecific arrangement of these compounds.

We did not find a stabilizing effect of CNT on the structure of iM at neutral pH being the effect of physical interaction between these two compounds. However, we found that deterioration of iM at neutral pH is at some moment hindered due to the presence of either guanine-rich supplementary strands (or Gq) or carbon nanotube. Anyway, the iM deteriorates quickly and spontaneously until it loses all hydrogen bonds in C:C Hoogsteen pairs. Afterwards the structure becomes rather static, but it cannot be called as i-motif since its characteristic factors (Hoogsteen pairs) do not exist anymore. Thus, the final conclusion related to the observed in literature stabilization of iM by carboxylated CNT is the following. Most probably it is due to proton transfer from carboxyl groups from functionalized CNT and formation of semiprotonated cytosines. Then, the iM-CNT complex becomes highly stable due to acid-base balance between carboxylated CNT and unprotonated cytosines. It is very likely that carboxylated nanotube also preferentially attack the Gq + iM part of DNA (or just cytosine-rich DNA fragments) with its tips, thus such chemical factor like proton transfer is highly possible to occur when protonated carboxyl residue approaches unprotonated cytosine. Then, stabilization or even formation of iM is very likely.

## Figures and Tables

**Table 1:** Interaction energy between fCNT and the whole iG, its Gq part, and iM part determined from the very last 4 ns of simulations. The energy is expressed in kJ mol^−1^.

System	a10	a20	n10	n20
iG	−833	−653	−959	−844
Gq	0	−241	−109	−347
iM	−38	−151	−327	−182

## Supplementary Materials

Supplementary materials can be found at https://www.mdpi.com/1422-0067/21/6/1925/s1.
