# Supplementary material for: Molecular Dynamics Study of the Interaction of Carbon Nanotubes with Telomeric DNA Fragment Containing Noncanonical G-Quadruplex and i-Motif Forms
Source: Int J Mol Sci. 2020 Mar 11;21(6):1925. doi: 10.3390/ijms21061925 (PMC7139311; doi:10.3390/ijms21061925)
Supplement: Supplementary file 1 [file ijms-21-01925-s001.zip › info.doc]

Supplementary material for

Molecular Dynamics Study of the Interaction of Carbon Nanotubes with Telomeric DNA Fragment Containing Noncanonical G-quadruplex and i-Motif Forms

Tomasz Panczyk*, Patrycja Wojton, Pawel Wolski

Institute of Catalysis and Surface Chemistry, Polish Academy of Sciences

ul. Niezapominajek 8, 30239 Cracow, Poland

These files can be used to analyze the associated configurations in a more detailed way. They can be directly visualized using vmd (<https://www.ks.uiuc.edu/Research/vmd/>), pymol (https://pymol.org/2/) or many other molecular visualization tools.

- Pdb files associated with the results shown in Figure 1:

Fig1_A.pdb – initial structure of the system

Fig1_B.pdb – final structure of the system after 40ns of equilibration

- Pdb files associated with the results shown in Figure 3:

Fig3_A.pdb – structure of a10 system after first REM run

Fig3_B.pdb – structure of a10 system after second REM run

Fig3_C.pdb – structure of a10 system after third REM run

- Pdb files associated with the results shown in Figure 4:

Fig4_a10.pdb – the optimal configuration obtained for the system a10

Fig4_a20.pdb – the optimal configuration obtained for the system a20

Fig4_n10.pdb – the optimal configuration obtained for the system n10

Fig4_n20.pdb – the optimal configuration obtained for the system n20

- Pdb files associated with the results shown in Figure 7, i.e. for systems obtained after
   removal of guanine residues from CNT:

Fig7_a10.pdb – configuration obtained for a10 system

Fig7_a20.pdb – configuration obtained for a20 system

Fig7_n10.pdb – configuration obtained for n10 system

Fig7_n20.pdb – configuration obtained for n20 system
